# Aerobiology from an Inactive Pyrite Mine: the Genome Sequence of the Airborne *Pseudomonas* sp. Strain L5B5

**DOI:** 10.1128/MRA.01029-21

**Published:** 2021-12-02

**Authors:** Jose L. Gonzalez-Pimentel, Irene Domínguez-Moñino, Valme Jurado, Ana Teresa Caldeira, Cesareo Saiz-Jimenez

**Affiliations:** a HERCULES Laboratory, Évora University, Évora, Portugal; b Instituto de Recursos Naturales y Agrobiologia de Sevilla (IRNAS-CSIC), Seville, Spain; University of Arizona

## Abstract

Pseudomonas sp. strain L5B5 is an antimicrobial-producing bacterium isolated from an air sample collected in a pyrite mine in Lousal, Portugal. Genomic analyses predicted genes involved in virulence factors. Here, we report the complete genome sequence of this bacterium, which consists of a circular chromosome with a length of 6,811,662 bp.

## ANNOUNCEMENT

The genus Pseudomonas is a very abundant group found in antagonistic environments; some interact with plants, some are useful as growth-promoting and biocontrol agents, and some are parasites, such as Pseudomonas aeruginosa and Pseudomonas syringae, two opportunistic pathogens for plants and animals (1). Pseudomonas are also found in subterranean environments, including mines, where some of them are able to remove heavy metals, which is useful for bioremediation (2, 3).

Mining is one of the oldest ways of producing and extracting minerals, and it has been intrinsically linked to the economic and social evolution of human beings. Lousal Mine (Grândola, Portugal) was closed in 1988 because the mined ores were no longer viable economically. Afterwards, within the environmental, social, economic, and heritage context, the mining area was remediated and rehabilitated in 2010, a geo-tourism center created, and a mining gallery opened to visitors (4).

The airborne Pseudomonas sp. strain L5B5 was isolated using a surface air system (Duo SAS, model Super 360). The sample was managed as described by Porca et al. (5). The culture medium was Trypticase-soy-agar (TSA; BD), and the culture plate was incubated at 28°C after picking a single colony. DNA extraction was performed using the Canvax HigherPurity bacterial genomic DNA isolation kit (Córdoba, Spain) with RNase treatment.

Genomic DNA was sequenced using both the NovaSeq 6000 (Illumina, USA) and SMRT RS II (PacBio, Menlo Park, CA) platforms. A TruSeq DNA PCR-free library was constructed using 150-bp paired-end short reads (Illumina); an SMRT library (insert size, 20 kb), constructed following the instructions for the Pacific Biosciences SMRTbell prep kit, was used for *de novo* sequencing. Default parameters were used for all software unless otherwise specified. The long reads were assembled using Canu v2.1.1 (6) and Circlator v1.5.5 (7) to circularize and orientate the chromosome. Pilon v1.24 (8) was employed for assembly improvement. Annotation was carried out using the NCBI Prokaryotic Genome Annotation Pipeline (9) and Sma3s v2 (10) with the “uniprot” flag. antiSMASH v6.0 (11), with the detection parameters in “strict” mode and all extra features on, was used to predict and annotate the secondary metabolite biosynthesis gene clusters. The Virulence Factors of Pathogenic Bacteria Database (VFDB), through Vfanalyzer, was used for virulence factors prediction (12). The Comprehensive Antibiotic Resistance Database (CARD), through Resistance Gene Identifier (RGI) software, was launched for prediction of the resistome (13). The closest relatives of L5B5 were identified using the JSpecies Web tool (14), and the average nucleotide identity was calculated using the BLAST (ANIb) and MUMmer (ANIm) algorithms.

The PacBio sequencing generated a total of 140,791 subreads, averaging 10,080 bp long, for a genome coverage of >208×, whereas the Illumina sequencing produced a total of 19,767,134 reads for a genome coverage of >435×. A tetra correlation search against the JSpecies database linked the LSB5 genome with the genomes of the type species Pseudomonas aestus CMM1215 and P. protegens CHA0 and with the biotechnologically useful strains P. protegens Cab57 and P. protegens Pf-5. The ANIb and ANIm values were below the 95% threshold suggested for species differentiation.

Table 1 provides a comparison of the relationship between L5B5 and related strains, as well as their functional characterization. L5B5 showed a more dynamic secondary metabolism, as well as an increased presence of virulence factors and genes involved in the resistome, with respect to the related genomes.

**TABLE 1:** Genome features and comparison between strain L5B5 and related bacteria

Strain	GenBank accession no.	GC content (%)	Genome size (bp)	Total no. of genes	No. of antiSMASH-predicted clusters	No. of VFDB mechanisms	No. of CARD hits
L5B5	CP084742	63.2	6,811,662	6,265	20	185	7 (strict)
*P. aestus* CMAA1215	AVOY00000000.1	63.8	6,658,235	6,380	19	169	4 (strict)
P. protegens Cab57	AP014522	63.34	6,827,892	6,236	15	175	4 (strict)
P. protegens Pf-5	CP000076	63.30	7,074,893	6,392	16	178	4 (strict)
P. protegens CHA0^T^	CP003190	63.39	6,867,980	6,252	15	178	4 (strict)

### Data availability.

The whole-genome shotgun project for Pseudomonas sp. L5B5 has been deposited at DDBJ/ENA/GenBank under the accession number listed in Table 1. The version described in this paper is the first version. The BioProject accession number is PRJNA769239, and the raw data have been deposited in the Sequence Read Archive (SRA) under accession numbers SRR16308509 and SRR16308510, for the long and short reads, respectively.
